# PROGRESS: the PROMISE governance framework to decrease coercion in mental healthcare

**DOI:** 10.1136/bmjoq-2018-000332

**Published:** 2018-07-16

**Authors:** Chiara Lombardo, Tine Van Bortel, Adam P Wagner, Emma Kaminskiy, Ceri Wilson, Theeba Krishnamoorthy, Sarah Rae, Lorna Rouse, Peter Brian Jones, Manaan Kar Ray

**Affiliations:** 1 Cambridgeshire and Peterborough NHS Foundation Trust, Fulbourn Hospital, Cambridge, UK; 2 Institute for Health and Human Development, University of East London, London, UK; 3 National Institute for Health Research Collaboration for Leadership in Applied Health Research and Care East of England, Cambridge, UK; 4 Norwich Medical School, University of East Anglia, Norwich, UK; 5 Department of Psychology, Anglia Ruskin University, Cambridge, UK; 6 Faculty of Health, Social Care and Education, Department of Adult and Mental Health Nursing, Anglia Ruskin University, Chelmsford, UK; 7 Faculty of Wellbeing, Education & Language Studies, The Open University, Milton Keynes, UK; 8 Department of Psychiatry, University of Cambridge, Cambridge, UK; 9 Addictions and Mental Health Services, Princes Alexandra Hospital, Metro South Health, Brisbane, Queensland, Australia

**Keywords:** patient satisfaction, patient safety, quality improvement methodologies, mental health, governance

## Abstract

Reducing physical intervention in mental health inpatient care is a global priority. It is extremely distressing both to patients and staff. PROactive Management of Integrated Services and Environments (PROMISE) was developed within Cambridgeshire and Peterborough NHS Foundation Trust (CPFT) to bring about culture change to decrease coercion in care. This study evaluates the changes in physical intervention numbers and patient experience metrics and proposes an easy-to-adopt and adapt governance framework for complex interventions.

PROMISE was based on three core values of: providing a caring response to all distress; courage to challenge the status quo; and coproduction of novel solutions. It sought to transform daily front-line interactions related to risk-based restrictive practice that often leads to physical interventions. PROactive Governance of Recovery Settings and Services, a five-step governance framework (Report, Reflect, Review, Rethink and Refresh), was developed in an iterative and organic fashion to oversee the improvement journey and effectively translate information into knowledge, learning and actions.

Overall physical interventions reduced from 328 to 241and210 across consecutive years (2014, 2015–2016 and 2016–2017, respectively). Indeed, the 2016–2017 total would have been further reduced to 126 were it not for the perceived substantial care needs of one patient. Prone restraints reduced from 82 to 32 (2015–2016 and 2016–2017, respectively). During 2016–2017, each ward had a continuous 3-month period of no restraints and 4 months without prone restrains. Patient experience surveys (n=4591) for 2014–2017 rated overall satisfaction with care at 87%.

CPFT reported fewer physical interventions and maintained high patient experience scores when using a five-pronged governance approach. It has a summative function to define where a team or an organisation is relative to goals and is formative in setting up the next steps relating to action, learning and future planning.

## Problem

Mind’s Mental Health Crisis report^1^ highlighted large variation in the annual rate of physical interventions (38–3000) between UK mental health trusts. Concerns about prone (face down) restraint and restraint-related injuries were also outlined. Patients who have been restrained report feeling stressed, fearful, angry, frustrated and confused.^2^ Even witnessing another patient being restrained can be distressing. Many of the feelings experienced by patients are shared by staff who also feel distressed.^2^ Having to use physical intervention, even as a last resort, is at odds with the care-driven practice. Staff report ‘It’s part of the job, but it spoils the job’^3^ (p. 215). Reviewing evidence of the effects of restraint, Cusack *et al*^4^ concluded that ‘restraint can be a form of abuse, its inappropriate use often being a consequence of fear, neglect and lack of using de-escalation techniques’ (p. 24). Coercion and restraint are mostly harmful and must stop being legitimised.^5^ There is an urgent need to challenge and address these practices as they represent gross human rights violations according to the Convention on the Rights of Persons with Disabilities.^6 7^

PROactive Management of Integrated Services and Environments (PROMISE)^8^ is a National Institute of Health Research Collaboration for Leadership in Applied Health Research and Care East of England supported project aiming to decrease coercion and restrictive practice within inpatient mental healthcare. It was launched within Cambridgeshire and Peterborough NHS Foundation (CPFT) in August 2013. Its aim was to reduce use of restraint and promote a more proactive and positive approach to care delivery. Decreasing restrictive practice was the primary objective; however, challenging custom and practice can make staff defensive, so the message to the front line was a positive reframe around enhancing patient experience. The initial focus was on prone restrains, and the goal was to eliminate its use within CPFT wards over a 3-year period from 2014 to 2017.

CPFT has 20 mental health inpatient wards, spread across all the psychiatric subdisciplines. Annually, it supports approximately 2000 inpatient care episodes for a population of 850 000 residents (2016 Census projections from 2011)^9^ of the county, along with regional and national referrals to its specialist units. The latter is of note as restrictive measures are more frequently used in specialist settings that can support highly distressed patients with significant safety concerns.

## Background

There has been a recent policy shift in the UK and internationally to reduce coercion in care.^10–16^ The WHO has made tackling human rights violations and promoting quality of care a key part of their Mental Health Action Plan 2013–2020.^16^

PROMISE was originally conceived in response to the Mind report and aimed to establish the scale of the problem within CPFT. Publication by the Department of Health in 2014 of ‘Positive and Proactive Care: reducing the need for restrictive interventions’^15^ provided further impetus, as PROMISE began operationalising the report recommendations. It was co-led by a clinician and a patient, an approach that was mirrored throughout the project. Two active advisory groups of patients and staff, supported a multidisciplinary team of clinicians, researchers and managers from a range of professional backgrounds. Within CPFT, restraint had been previously classed as a ‘necessary evil’^2^ (p .500) used to maintain safety and meet patient treatment needs. Engagement of modern matrons, ward managers and consultant psychiatrists was vital in highlighting to front-line nurses, occupational therapy staff and healthcare assistants, how extremely traumatic and dehumanising restraints are.

The PROMISE research team carried out a scoping review of 60 studies of restraint reduction in mental healthcare from 2004 to 2014.^17^ Interventions were mostly multifaceted, and the majority focused on reducing mechanical restraint (rarely used in the UK), with most reporting a reduction in restraint use. Across the 60 studies reviewed, aspects of the described initiatives fell into five broad categories: proactive care, organisational development, empowerment, communication, and relationships and reviewing practice (online supplementary table 1). While the review highlighted interventions that were informative to healthcare providers, given the methodological limitations and non-UK focus, this area needed further exploration. So, the PROMISE research team conducted a qualitative study with CPFT mental health patients and staff that explored their suggestions for reducing restraint^18^ in the UK context. Findings centred on four key themes: improving communication and relationships between staff/patients, making staff-related changes, improving ward environments/spaces and having more activities.

10.1136/bmjoq-2018-000332.supp4 Supplementary data

## Measurement

Given the primary target was eliminating prone restraints, restraint numbers became the principal outcome measure. An audit of restraint incidents across the 20 mental health inpatient wards in 2014 provided an annual baseline count of 328. However, it revealed that incidents could not be categorised into subtypes of restraint, as such detail was not consistently captured. However, at this time, most restraint ended in prone restraint: it was taught as the intervention of choice in CPFT’s Prevention and Management of Violence and Aggression (PMVA) training and was thus routine practice.^19^ Given PROMISE’s aspirations of moving towards less restrictive practice, the organisation’s incident reporting system (DATIX) was subsequently refined to record restraint type used to gauge shifts in change of practice. Additional refinements included: recording prone restraint duration, reason for restraint and debrief following restraint.

There were concerns that measuring impact by a sole focus on restraint numbers would not energise staff. It can be misleading as it is a crude metric that is easily skewed by the care needs of individual patients needing repeated restraints. This can be quite demotivating for staff, as they may feel that in spite of their best efforts, they are not delivering on their goal. Positive engagement in improving patient experience is far more engaging than decreasing restraint numbers. Given these limitations, patient experience scores were also considered. Inpatients were asked to complete an online anonymous satisfaction questionnaire. Examples of items interrogated are listed in online supplementary table 2 and were based on Care Quality Commission’s inpatient survey.^20^

10.1136/bmjoq-2018-000332.supp5 Supplementary data

## Design

A complex multifaceted quality improvement intervention was implemented as part of PROMISE. It drew on the scoping review^17^ and the qualitative study mentioned above.^18^ Five overlapping categories emerged across the two studies, and initiatives were coproduced based on these findings between managers, clinicians, patients and carers. Some examples include:

- In ‘Proactive Care’, over a hundred staff were trained in sensory integration,^21^ and sensory rooms and gardens were set up on wards to decrease reliance on restraint. Initiatives like ‘No audit’,^22^ which encourages a more ‘can do’ attitude around individualising care, were central in changing the nature of day-to-day interactions. Environments on wards were changed from being sparse and clinical to a more healing and ‘homely’ environment.
- Within ‘Organisational Development’, considerable investment was made in leadership training programmes with special emphasis on ward managers and modern matrons. New nurse specialist posts were created on every ward to enhance nursing leadership.
- In ‘Empowerment’, staff were encouraged to map out the therapeutic day and make small changes with potential for significant impact on the patient journey. These were collated through ‘Mapping Frontline Initiatives’ programme^23^ and systematically shared through quarterly wider leadership events.
- In ‘Communication and Relationships’, the PMVA training was revamped with much greater focus on communication, de-escalation and building therapeutic alliance. Many front-line initiatives, such as having lunch with patients, morning community meetings and caring for carers, related to building relationships.
- For ‘Reviewing Practice’, newly appointed nurse specialists played a pivotal role in supporting a culture of reflection and appreciative enquiry. Regular debriefs were introduced, and data relating to restraint incidents and patient experience were shared within a monthly cycle within each ward. There was a focus on performance, but the approach was one of curiosity and help rather than summative judgement.

The overall goal of this multifaceted intervention was to create leadership at every level that reinforced the belief that ‘the patient is not in the way, patient is the way’.

## Strategy

To manage the improvement initiatives within PROMISE, and the complexity related to the human dimensions of change, governance became essential. PROactive Governance of Recovery Settings and Services (PROGRESS), a governance framework, was developed to manage improvement of this nature and scale. We used a naturalistic mixed methods approach that was both summative and formative. The framework calls for five key actions, each of which has a specific objective in mind and provides guidance on temporal frequency (online supplementary table 3).

- Report: daily reporting was embedded into business as usual with a balance between the amount of time spent reporting and providing patient care. For data to be helpful, the incident reporting system needed to be fit for purpose and intuitive. It needed to capture the essential data relating to ‘how many’ incidents and qualitative information regarding the antecedents through meaningful postincident debriefs with patients and staff. This information enabled the ward team to make real-time changes to patients’ individual care plans. Good reporting practices translated data into usable information.
- Reflect: weekly reflection within the multidisciplinary team helped clinicians share and learn from the incidents or near misses and from what worked well. Diverse views were captured and reflected on. In particular, carefully considering the patient perspective highlighted missed opportunities and informed future care provision for both individuals and care settings. Non-judgmental mindful reflective practice helped translate information gathered through reporting into replicable knowledge.
- Review: monthly review of reported summative information started in March 2015 and supported the whole organisation to stay on track with the improvement trajectory. The reviewed metric changed over time as the improvement cycle progressed. Metrics considered included: incident numbers, incident type, wards involved, time of the day of incidents, reason (eg, aggression, self-harm, absconsion and adherence to medication) and trends. Patient representation, along with staff from different care settings, helped with problem solving when a metric was lagging or a particular team had specific difficulties. Review meetings translated summative knowledge into concrete contextual formative actions.
- Rethink: quarterly meetings, from January 2014, with the wider leadership helped maintain momentum for the improvement agenda. It increased exposure to new ideas and initiatives and allowed learning from each other’s success and challenges. Desired outcomes from monthly review meetings resulted in front-line initiatives/actions that were contextualised to the care setting. Different staff groups approached the same issue (eg, improving patient experience) in different ways. These initiatives were captured, collated, celebrated and shared in Rethink meetings. They helped banish innovation islands and ensured that learning was shared and successes were replicated across the organisation and beyond.
- Refresh: annual business planning cycles were used to refresh goals and propose trajectories. Work plan along with resource outlay to deliver key performance indicators (KPIs) targets were agreed. KPIs included physical intervention numbers, measures of patient and staff experience and clinical effectiveness measures. To enthuse staff and bring to life the stories behind the KPIs, the new plan was launched in a celebration event (signing of PROMISE Charter – 9 October 2015 involving patients, carers and staff from CPFT and statutory and non-statutory partner organisations).

10.1136/bmjoq-2018-000332.supp6 Supplementary data

## Results

### Physical intervention

Restraint data were extracted from the Trust incident reporting system and compiled into monthly reports for the Positive and Proactive Care Review Group. The baseline figure for total number of restraints for 2014 (calendar year) was 328.

As explained above, during the first quarter of 2015, CPFT’s incident reporting system was refined; thus, the next two 12-month cycles map on to the financial year (April–March).

**Figure 1:**
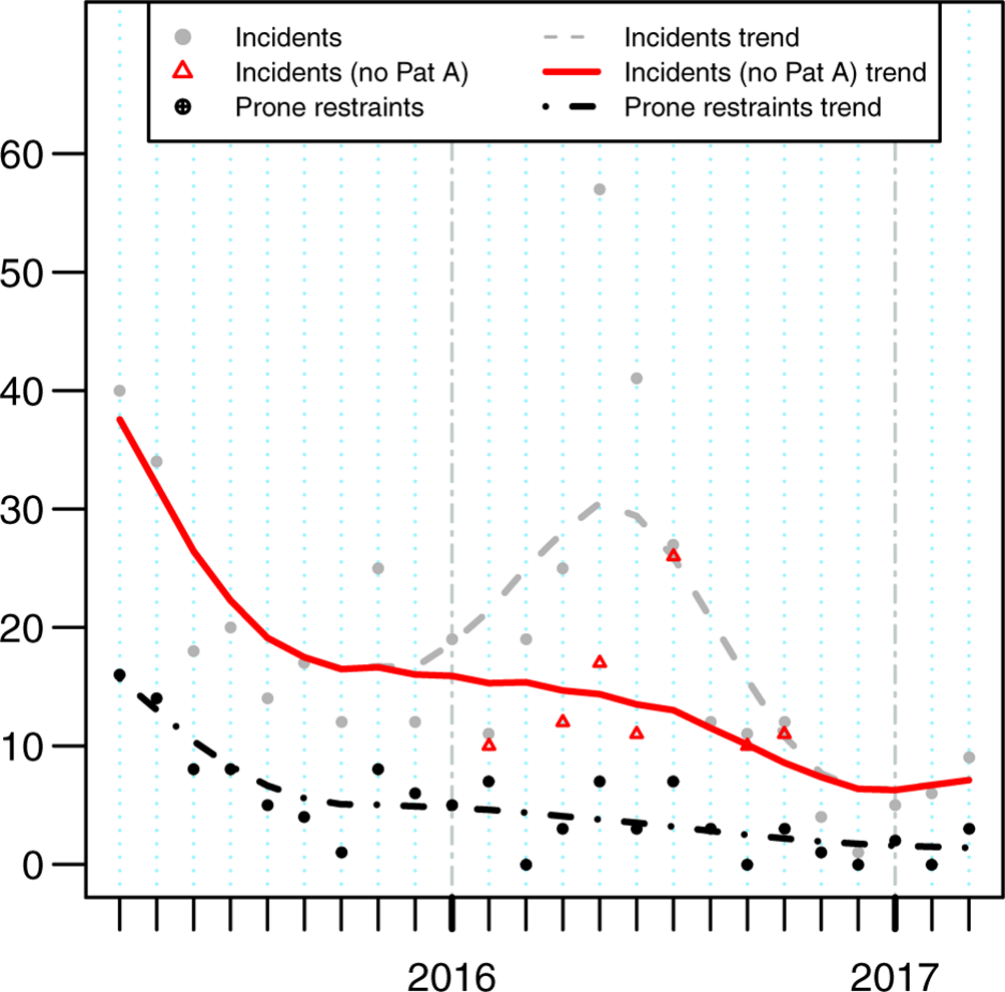
Restraint incidents (with and without a patient who was restrained many times; see text), and number of incidents involving prone restraint by month. Smoothed trend lines are produced by Friedman’s ‘super smoother’.

From table 1 and figure 1, we see a broad decreasing trend in both the number of physical intervention and prone restraint incidents, particularly in comparison with the 2014 incident numbers. Tests of a polynomial cubic trend within Poisson regression models for the both number of physical intervention incidents (excluding patient A; see below) and prone restraints were statistically significant (χ²(3)=78.86, p<0.0001 and χ²(3)=49.13, p<0.0001, respectively).

**Table 1:** Physical intervention numbers by ward type across 2014, 2015–2016 and 2016–2017

Ward type (number)		Prone restrains*		Full physical interventions		
		2015–2016	2016–2017	2014	2015–2016	2016–2017
Generic adult wards	Assessment (2)	1	3	6	11	12
	Treatment (3)	19	14	82	55	51
	Recovery (2)	6	4	19	10	14
Specialist wards	Psychiatric intensive care (1)	13	2	33	43	8
	Personality disorder (1)	3	1	57	11	1
	Low secure (1)	1	3	7	3	9
	Eating disorder (1)	0	3	1	1	86†
	Learning disabilities (2)	19	0	18	51‡	9
	Older adults (4)	9	1	5	29§	7
	Child and adolescent (3)	11	1	100	27	13
	**Total across wards**	**82**	**32**	**328**	**241**	**210**

*2014 prone restrain data are not available as recording subtypes was not mandatory in 2014.

*†Relates to a single patient.

‡41 of 51 physical Interventions relate to a single patient (more details in online supplementary figure 1 and table 4).

§Impact of enhanced reporting on older adult wards.

Prone restraints, a primary focus particularly early on in PROMISE, dropped from 82 (2015–2016) to 32 (2016–2017), a reduction of 61%. Restraint type used was not consistently recorded, but we expect the vast majority of the 328 incidents to be prone as this was standard practise then. Thus, we expect there was an even bigger proportional reduction from 2014 to 2015–2016.

Restraint incidents decreased from 328 (2014), 241 (2015–2016) to 210 (2016–2017) successive reductions of 27% and 13%. However, during 2016–2017, we had an example of an individual patient significantly impacting figures: the spike in May 2016 (Figure 1) was driven by patient A (online supplementary table 4 and figure 1) struggling with a severe eating disorder and a precarious body mass index; every feeding attempt was crucial but, unfortunately, required restraint to be successful. Excluding this patient, the total for 2016–2017 would have reduced to 124 and would have represented a 48% reduction on the previous year (2015–2016).

The overall reduction in restraint incidents shows the reduction in prone restraints did not result in a shift to other forms of full physical intervention (eg, supine, kneeling and sitting). A similar reduction over time was noted in the number of individuals being restrained (online supplementary figure 2).

10.1136/bmjoq-2018-000332.supp2 Supplementary data

The most marked improvements were observed in the specialist wards (table 1). For example, the learning disability (intellectual disability) inpatient unit had a large reduction in restraints from 51 (2015–2016) to 9 (2016–2017), an 82% reduction, and prone restraints from 9 to 2. A similar reduction was seen in psychiatric intensive care unit: 43 (2015–2016) to 8 (2016–2017), an 81% reduction, and prone restraints from 13 to 2. The specialist personality disorder unit was prone restrain free for 23 of the 24 months, and full physical intervention numbers fell from 11 to 1.

By March 2016, 16 of the 20 wards had a 6-month stretch of zero prone restrains, which increased to 18 out of 20 by March 2017. All 20 wards had a 4-month stretch of zero prone restrains in each year. For full physical intervention, by March 2016, 17 of the 20 wards had a 3-month stretch of no full physical intervention, which increased to 20 by March 2017.

### Patient experience

**Figure 2:**
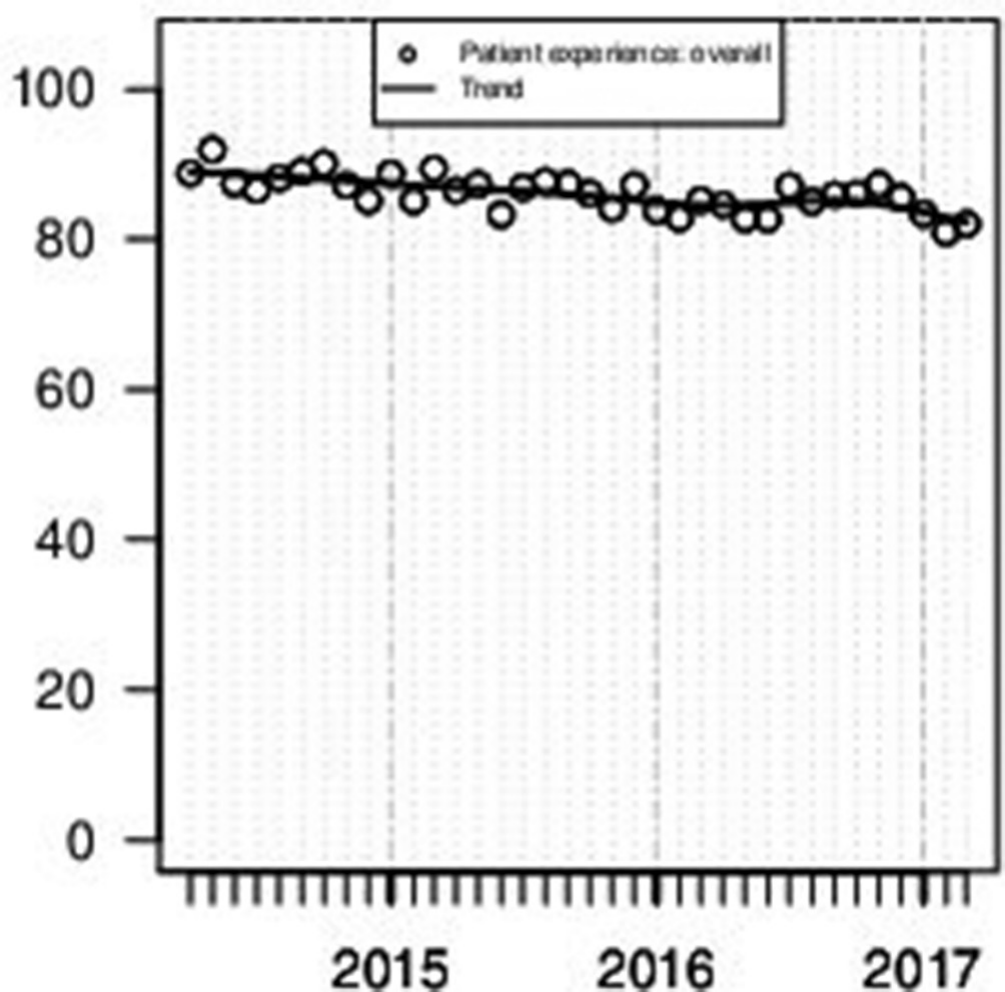
Monthly ‘Overall Performance’ for patient experience ratings (range between 0 and 100). Overall performance is a summary measure based on responses to individual items. Smoothed trend line produced by Friedman’s ‘super smoother’.

Figure 2 shows the monthly ‘Overall Performance’ (a summary across items) of patient experience from April 2014 to March 2017. An average score of 87% for the whole period was recorded across 4591 surveys (approximately 75% of inpatients). There is a small significant downward linear trend (linear trend test in a linear regression model: F_34,35_=28.63, p<0.0001). A key driver of this trend is thought to be a change in time of surveying to capture the experience of acutely unwell patients.

The top three items are attitudinal (staff polite and friendly: 98%; admission welcoming: 97%; respect and dignity maintained: 96%) and are followed in rank order by action oriented measures (medication purpose explained: 94%; weekday activities supported: 93%; have a care plan: 93%). The individual patient experience items are plotted by month in online supplementary figure 3 and reported by year in online supplementary table 2. To combine attitudes and actions, in August 2016, a new question was introduced to elicit whether patients felt involved in their care/treatment discussion. A 98% rating across 964 surveys on this item shows the effort staff made to involve patients in their care irrespective of their voluntary or involuntary status (online supplementary table 5).

10.1136/bmjoq-2018-000332.supp3 Supplementary data

10.1136/bmjoq-2018-000332.supp8 Supplementary data

### Relationships between restraint numbers and patient experience

Patient experience data are completely anonymous and cannot be linked to individuals restrained. The correlation between the detrended (series values minus Friedman’s ‘super smoother’ fit, for April 2015–March 2017) overall patient experience and restraint incidents (excluding patient A) is 0.14, a small and non-significant correlation (p=0.5235). It is necessary to detrend the series to reduce the risk of spurious correlations caused by trend.

### Lessons and limitations

At the heart of PROMISE is an alternative approach to governance. Initially, as an organisation, we were focused on targets and trajectories. These are important tools for measuring progress. However, engagement exercises highlighted that behaviours and practices that we were endeavouring to change through KPIs were only the visible tip of an iceberg. One could hit the target but completely miss the point. What kept restrictive practice embedded was the existing culture, the invisible bulk of the iceberg. We made a decision early on to grapple with culture and address it head on in order to avoid a situation where as soon as one target or behaviour is addressed another would emerge. This is tiring for leadership and demotivating for front-line staff. So cultural change^24^ was needed at two levels: first, front-line culture needed changing to reduce the use of restraint, and second, a wider cultural shift from focusing from ‘target chasing’ to a more holistic improvement mindset. We expected an improvement in the second would contribute to increased gains in the first.

The PROGRESS framework aimed to manage this transition of a target-driven governance mindset of ‘have to’ to an outcome based ‘want to’ approach through adopting temporal sequencing of the five actions. The former results in staff who are overwhelmed with delivering KPIs and are feeling defensive if they are failing. The latter empowers and enthuses staff to find novel solutions to challenges and draw on the existing expertise of patients and carers. The five actions of PROGRESS (Report, Reflect, Review, Rethink and Refresh) grapple with culture and create a mindful learning environment and ensure that the story of better patient care remains at the forefront. This is particularly important when addressing restrictive practice. Physical intervention does not sit well with core staff values of caring and was considered a necessary evil.^2^ Generally, staff are acting out of welfare or safety concerns and are at the receiving end of overwhelming distress in a patient. It is a highly emotive subject, and a governance framework that makes staff feel as if they are failing or being criticised will only make them defensive.^25^ Antecedents to restraint vary considerably, and defensive staff do not go looking for creative individualised solutions. On the contrary, energies get diverted to rationalising what is happening. Thus, a fine balance needs to be struck to ensure that the issue is not diluted, but simultaneously staff engage and own the improvement agenda.^2 18^ In PROGRESS, this is achieved through a natural counterbalance of summative data in the report and review actions and the formative non-judgemental curiosity of reflect and rethink steps. In fact, there is considerable overlap in the attitudinal aspects of all five steps.

The adult treatment wards provide an interesting case study. There were a large number of initiatives that the wards embraced that resulted in very high patient experience scores and a 33% decrease in 2014 restrain numbers, but there was limited decline in the next year. A careful analysis showed that most of the patients being restrained were those suffering from severe mania and needed restrain for their own safety (ie, to prevent absconsions or to protect their dignity). An atmosphere of more light (non-judgemental genuine curiosity) and less heat (to stop staff feeling defensive and demotivated) has been maintained throughout, so that the wards did not engage in target chasing (restraint numbers). Two key pieces of work are being undertaken. First, a study has been launched to assess whether advanced psychological formulation of predisposing, precipitating, perpetuating and protective factors can break the cycle of restraint in this group of very unwell hard to engage patients. Second, further qualitative analysis of electronic patient records of patients with repeat restraints to establish missed opportunities for early identification and intervention relating to escalating distress.

PROMISE proposes a complex multifaceted intervention, where culture is addressed as a means to change the nature of day-to-day interactions. Thus, it is difficult to attribute causality to any specific aspect of PROMISE. However, PROGRESS, with an alternative mindset to governance, provides a framework in which staff and patients can feel empowered to coproduce a new care culture that moves practice away from coercion. It creates a therapeutic milieu in which practitioners can provide a caring response to distress, irrespective of its source or height.

The framework breaks the transformation journey into bite-sized chunks and makes it generalisable (online supplementary table 3). Different organisations will be at different stages in their journey of setting up systems and processes around quality assurance. Therefore, they may vary in the sophistication of their incident reporting systems, or culture of reflective practice, or ward to board accountability, or systems to support learning. Before adopting and/or adapting PROGRESS, we recommend a baseline exercise. Some quantitative data are needed to establish the scale of the problem relating to restrictive practice (eg, numbers of seclusion, restraints and rapid tranquilisation). If such data do not exist due to the absence of an incident reporting system, or it is not readily accessible, this would need to be prioritised.

Establishing a baseline for patient experience is equally important. Embedding systems and processes for routine reporting and reflection on patient experience and physical intervention data provided creative avenues to address restrictive practice. The monthly reviews were not just summative but also resulted in formative improvements to the survey (online supplementary table 5) and patient experience initiatives. The quarterly Rethink meetings with the wider leadership were focused on collating and sharing successes and challenges from these initiatives.

A proposal to raise the bar and capture the experience of patients when acutely unwell by changing the time of survey administration was finalised in the October 2015 annual Refresh (PROMISE Charter Event). In 2014–2015 surveys were conducted at discharge; subsequently, patients were surveyed as soon as clinically appropriate. This change is thought to be a key driver for the small downward trend. Additionally, following the national trend, CPFT has reduced its number of adult acute beds. Overall, this means that the average admitted patient will be more distressed and have higher severity of illness, resulting in lower patient experience scores. This effect is thought to be particularly acute in CPFT, with its reduction in beds of 44% compared with the national average reduction of 17% (Kay Ray *et al*, 2018).^26^ Within the context of these factors, restricting a change in patient experience to only a small decline is a substantial achievement. These issues highlight how patient experience is impacted by a great many internal and external factors: it is perhaps not surprising to find only a small relationship between restraint numbers and patient experience.

PROGRESS was supported by two active advisory groups (patients and staff) who provided in-depth insight into custom and practice. Coproduction was at the heart of this project, and the early involvement of patients and front-line staff played a significant part in the way governance structures iteratively evolved. As evidenced in online supplementary table 6, PROMISE had a strong research strand from its inception to gather baseline cultural information.^2 17 18^ Research helped get the story out to the front line and created a stance of curiosity with a genuine desire to know rather than provide judgement. Contextual organisational research can supplement the foundations of a governance framework.

10.1136/bmjoq-2018-000332.supp9 Supplementary data

## Conclusions

PROMISE is a complex intervention to bring about culture change in the context of restrictive practice and the use of force in mental health settings. Over a 3-year period, CPFT had a remarkable drop in incidents of prone restrains and all forms of restraint as well as registering high patient experience scores. The five actions of PROGRESS (Report, Reflect, Review, Rethink and Refresh) provide a framework through which a sensitive subject, like coercion in care, can be addressed in a decisive manner. It is suited to a naturalistic approach to evaluation and is sensitive to both the improvements and unintended consequences of the transformation work. It is not designed to support claims of causality to specific aspects of the implemented change; rather it can help overcome the challenges in transferring learning and replicating a multifaceted intervention like PROMISE in another organisation with aspirations similar to those of CPFT.

10.1136/bmjoq-2018-000332.supp1 Supplementary data

10.1136/bmjoq-2018-000332.supp7 Supplementary data
