# Supplementary material for: PROGRESS: the PROMISE governance framework to decrease coercion in mental healthcare
Source: BMJ Open Qual. 2018 Jul 16;7(3):e000332. doi: 10.1136/bmjoq-2018-000332 (PMC6059331; doi:10.1136/bmjoq-2018-000332)
Supplement: Supplementary data [file bmjoq-2018-000332supp004.docx]

Supplementary Table 1: Breakdown of restraint reduction initiatives across the 60 studies reviewed.

| **PROACTIVE CARE** | ***Patient activities*** | Puzzles  Sewing  Sport/Exercise  Occupational Therapy | Make a photo album  High tea  Sensory modulation/comfort rooms  Multi-purpose activity room |
| --- | --- | --- | --- |
|  | ***Staff training*** | De-escalation  Trauma-informed  Recovery-oriented | Crisis intervention training  Alternatives to restraint |
|  | ***Assessment and planning*** | Discharge planning meetings  Daily treatment plans  Psychiatric advance directives  Advance crisis management plans | Violence assessment and monitoring  Behaviour plans  Identification of restraint-prone patients  Multi-disciplinary care meetings |
|  | ***Specific initiatives*** | Medical interventions  Patient massage  Positive reinforcement  Availability of a quiet room | Early intervention/management  Restraint reduction kit  Stress/anger management for patients |
|  | ***Flexibility*** | Relaxing rules (e.g. off-unit privileges) | Less intense special observations |
|  | ***Environment*** | Give patients a change of scenery  Relocate wards to non-institutional environments  Recovery-oriented environments | Play music on wards  More natural light on wards  More open areas on wards |
| **ORGANISATIONAL DEVELOPMENT** | ***Leadership*** | Leadership towards organisational change  Strong leadership | Form a restraint reduction task force  Clearly define staff roles/hierarchy |
|  | ***Organisational culture*** | Hold open forums for staff  Change employee selection process | Change employee orientation process  Provide regular training |
|  | ***Organisational mission, policies, and goals*** | Organisational goal setting  Define force as treatment failure  Change mission/policies to reflect commitment to  recovery | Shorten interval between mandatory renewal  orders  Constantly monitor patient during restraint |
|  | ***Changes to staffing*** | Change ward staff  Increase nurses per shift | Introduce a dedicated inpatient psychiatrist  Hire peer employees |
| **EMPOWERMENT** | ***Empowering staff*** | Staff stress reduction training  Reflective space for staff  Increased roles in decision-making  Opportunity to develop/implement own initiatives | Open forums for staff  Exploration of staff concerns  Staff contest (creative alternatives to restraint)  Incentive system for staff |
|  | ***Empowering patients*** | Increased role in decision-making  Increased role in treatment planning  Strength-based treatment | Peer support  Increased role in incident reviews |
| **COMMUNICATION & RELATIONSHIPS** | ***Patients and staff*** | Improved communication  Clearly written patient rights/expectations  Frequent patient checks/questions  Collaborative problem solving  Collaborative models of care  Patients and staff going walking together | “Risk-sharing” partnerships  Increased contact time  Staff identification boards  Positive communication from staff  Recovery language  Patient comment forms |
|  | ***Leadership and staff*** | Better communication pathways  Mentoring  Communicate organisational goals | Regular and positive feedback to staff  Regular interactions to support a working  relationship |
|  | ***Between staff*** | Staff “round tables” to discuss ideas for reducing  restraint  Consult each other prior to approaching patients in  crisis | Accountability  Multidisciplinary communication  Conferences to share ideas/initiatives |
|  | ***Staff training*** | Empathy and listening skills | Verbal skills training |
| **REVIEWING PRACTICE** | ***Use of restraint data*** | Tracking and trending incidents  Data to inform practice  Evaluate what works | Feedback data to staff  Inform authorities |
|  | ***Debriefing*** | Non-punitive  Supportive  Within 24 hours of incident | Includes patient  Includes care team |
|  | ***Review*** | Critical incident review  Rapid response team review | Crisis response team |
