# Supplementary material for: PROGRESS: the PROMISE governance framework to decrease coercion in mental healthcare
Source: BMJ Open Qual. 2018 Jul 16;7(3):e000332. doi: 10.1136/bmjoq-2018-000332 (PMC6059331; doi:10.1136/bmjoq-2018-000332)
Supplement: Supplementary data [file bmjoq-2018-000332supp005.docx]

Supplementary Table 2: Patient experience survey results: values are percentages of surveyed patients agreeing with each item (Yes/No). *Responses to ‘Care rating and Food rating’ are on a Likert scale – this is converted to a percentage. Overall performance is a summary measure based on responses to individual items.

| **Patient experience item** | **Overall** | **By year** | | |
| --- | --- | --- | --- | --- |
|  | **2014-17 (n=4,591)** | **2014-15 (n=1,726)** | **2015-16 (n=1,447)** | **2016-17 (n=1,418)** |
| Accommodation needs met | 90 | 91 | 90 | 88 |
| Admission welcoming | 97 | 98 | 96 | 96 |
| Care choices supported | 89 | 92 | 88 | 87 |
| Care plan meeting organised | 92 | 94 | 91 | 89 |
| Employment needs supported | 75 | 78 | 78 | 66 |
| Feel safe | 84 | 86 | 84 | 83 |
| Financial needs supported | 83 | 83 | 83 | 82 |
| Food rating* | 65 | 65 | 64 | 67 |
| Have a care plan | 93 | 96 | 93 | 88 |
| Healthy living supported | 78 | 78 | 78 | 76 |
| Key professional known | 89 | 91 | 88 | 89 |
| Medication purpose explained | 94 | 95 | 94 | 91 |
| Medication side effects explained | 75 | 78 | 73 | 71 |
| Medication views | 85 | 87 | 85 | 81 |
| Care rating* | 87 | 89 | 86 | 84 |
| Respect and dignity maintained | 96 | 97 | 96 | 95 |
| Staff polite and friendly | 98 | 98 | 97 | 97 |
| Understand care plan | 84 | 87 | 83 | 80 |
| Weekday activities supported | 93 | 95 | 90 | 92 |
| Weekend activities supported | 72 | 75 | 70 | 71 |
| **Overall performance** | **87** | **88** | **86** | **85** |
