# Supplementary material for: PROGRESS: the PROMISE governance framework to decrease coercion in mental healthcare
Source: BMJ Open Qual. 2018 Jul 16;7(3):e000332. doi: 10.1136/bmjoq-2018-000332 (PMC6059331; doi:10.1136/bmjoq-2018-000332)
Supplement: Supplementary data [file bmjoq-2018-000332supp006.docx]

Supplementary Table 3: The Five Steps of PROGRESS.

| ***Report daily: data to information***   - Create a culture of daily reporting and set up systems to support it. - Establish what is available and what is needed and categorize requirements into essential, desirable and aspirational - Measuring both from a summative (to pass judgment on impact) and formative (to suggest ways to improve the portfolio of initiatives) perspective is helpful. - Seek usefulness rather than perfection as the goal is improvement not to develop a measurement system. - A mindset of more light and less heat can be supported by metrics that measure serious incidents as a proportion of all incidents. They set up a virtuous cycle of improving reporting and a culture of openness and curiosity. |
| --- |
| ***Reflect weekly: information to knowledge***   - If we just manage what we measure then we will end up doing what we count, not what counts. Reporting without reflection only provides numbers without narrative. - From incidents to antecedents: Using the patient’s version of events in guided reflection sessions can open up new avenues and help identify missed opportunities. - In the face of overwhelming distress in a patient, staff often question ‘what is the matter with you’. Reflective practice can help staff transition to a positive and proactive mindset of ‘what matters to you.’ - A shift from tackling antecedents to enhancing patient experience which encourages brainstorming for coproduction of innovative solutions. |
| ***Review monthly: knowledge to action***   - Often available data is not used because it is inaccurate, incomplete, late or inconsistent and as it is not used no one bothers to make the information accurate, complete, timely or consistent creating a vicious cycle. - This information vicious cycle can be broken by making existing data the basis for decisions and actions in a sensitive fashion. The frontline teams ultimately hold the key to improving data quality. - Review translates knowledge into action and guides services from established baseline to agreeing targets and trajectories against which progress is monitored. - It is essential to complement restraint data with Patient Experience measures as they will provide a balanced view of care provision and are more difficult to skew. - In lines with appreciative enquiry, ward and directorate leadership should feel able to hold the other to account on commitments made in the previous monthly cycle. - The metrics under scrutiny will shift and evolve over time and will depend on the actions that were due to be carried out within the month. |
| ***Rethink quarterly: action to learning***   - A culture of empowerment and making innovation everyone’s business can be supported by collating and showcasing success in a consistent fashion. - Time spent in rethinking patient care / pathways is a spend to save initiative and more than makes up in time lost in reacting to incidents. - These quarterly events are essential to keep leadership enthused and mindfully responding to ever evolving standards to aspire to, on the improvement journey. - Success breeds success and celebrating new standards and the achievements of the innovators will result in ideas springing from all sectors. |
| ***Refresh annually: learning to planning***   - Based on the progress, quality assurance business plans need refreshing annually. - New stretch targets and trajectories against key measures should be thought through in an iterative fashion with inpatient leadership to increase ownership. - A balance of KPIs across patient safety, clinical effectiveness, patient experience and staff engagement will be needed to change care culture and decrease restrictive practice. - Key initiatives regarding how to deliver on the metrics along with resource outlay should be an upfront discussion. - The story behind the refreshed plan ought to be shared at an annual celebration event in which successes are celebrated, challenges are acknowledged and the next phase of the journey is talked through with frontline staff. |
