# Supplementary figures and images for: PROGRESS: the PROMISE governance framework to decrease coercion in mental healthcare
Source: BMJ Open Qual. 2018 Jul 16;7(3):e000332. doi: 10.1136/bmjoq-2018-000332 (PMC6059331; doi:10.1136/bmjoq-2018-000332)

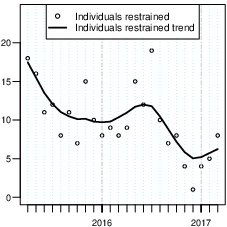

Supplement: Supplementary data [file bmjoq-2018-000332supp002.jpg]

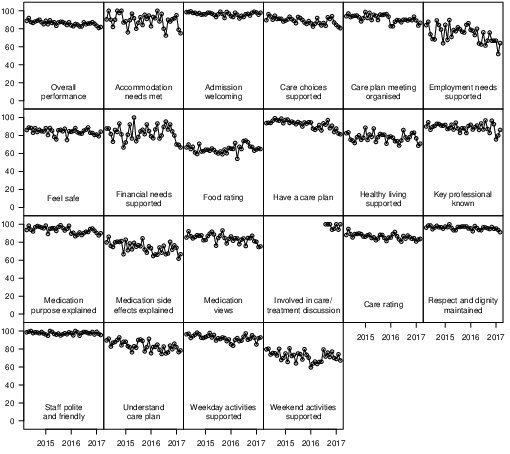

Supplement: Supplementary data [file bmjoq-2018-000332supp003.jpg]

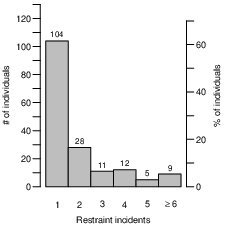

Supplement: Supplementary data [file bmjoq-2018-000332supp001.jpg]
