# Supplementary material for: PROGRESS: the PROMISE governance framework to decrease coercion in mental healthcare
Source: BMJ Open Qual. 2018 Jul 16;7(3):e000332. doi: 10.1136/bmjoq-2018-000332 (PMC6059331; doi:10.1136/bmjoq-2018-000332)
Supplement: Supplementary data [file bmjoq-2018-000332supp008.docx]

Supplementary Table 5: Percentages of patients who felt that they were involved in care/treatment discussion

| Month | Involved in care/treatment discussion (%) | No of surveys |
| --- | --- | --- |
| Aug-16 | 100 | 104 |
| Sep-16 | 100 | 113 |
| Oct-16 | 100 | 132 |
| Nov-16 | 94 | 120 |
| Dec-16 | 95 | 128 |
| Jan-17 | 100 | 127 |
| Feb-17 | 94 | 120 |
| Mar-17 | 100 | 120 |
|  | **98** | **964** |
