# Supplementary material for: PROGRESS: the PROMISE governance framework to decrease coercion in mental healthcare
Source: BMJ Open Qual. 2018 Jul 16;7(3):e000332. doi: 10.1136/bmjoq-2018-000332 (PMC6059331; doi:10.1136/bmjoq-2018-000332)
Supplement: Supplementary data [file bmjoq-2018-000332supp009.docx]

Supplementary Table 6: Setting up the PROMISE journey.

| July 2013: Beginning of the PROMISE journey with the formation of a working group | | | | |
| --- | --- | --- | --- | --- |
| **Time Line** | **Activity** | **Type** | **Key Insight / Finding** | **Actions / Relevance to Governance** |
| Dec 2013 | Completion of one month in depth audit of all physical interventions in the trust | Audit | Restraint is the tip of the iceberg. Mindset shift from incidents to antecedents to elicit missed opportunities that would change the trajectory of events | Daily reporting on patients experience.  Reflection following incidents and near misses |
| Jan 2014 | Engage the wider leadership of the adult directorate at the quarterly event and seek a steer for the road ahead | Publicity | Focusing on enhancing patient experience rather than just decreasing restraint numbers will be energising and empowering for staff | Monthly review meetings incorporating patient experience (PE) data.  Quarterly rethink meetings sharing initiatives to improve PE. |
| Feb – June 2014 | Systematic review into restrain reduction programmes over a 10 year period | Research | There was a concerning lack of studies which were relevant to the UK setting and the vast majority of interventions were multi-faceted which made it difficult to compare different approaches. | Development of a qualitative study in the UK setting incorporating positive and proactive care suggestions from patients and staff who have been involved or witnessed restraint. |
| July – Dec 2014 | Mapping Frontline Initiatives | Research | There is tremendous innovation at the frontline and collating and sharing these initiatives in a systematic way can act as a catalyst for the desired outcome. | Quarterly rethink events that can help to disseminate new and exciting initiatives. |
| Aug 2014 – June 2015 | Qualitative Research into staff and patient experience of physical intervention and suggestions for positive and proactive care | Research | Confirmed previous insights around focusing on antecedents and improving patient experience. Emphasised the need for a mindset shift from just focusing on inpatient care to missed opportunities in community care. | Reflective practice needs to incorporate both inpatient and the community pathways and how care in one area influences the other. |
| Jan 2015 | Audit of all physical interventions in 2014 and review of our incident reporting system | Audit and Database Improvement | We manage what we measure. | Fit for purpose reporting system is essential – Datix was overhauled in line with aspirations and reporting requirements and monthly spreadsheets started to get compiled |
| March 2015 | Trust wide Positive and Proactive Care group set up | Service Improvement | If we just manage what we measure then we will end up doing what we count, not what counts. | Monthly review meeting needs to go beyond the numbers and explore the hows and whys so that the governance discussion is summative and formative e.g. Shifting the focus in training. |
| Oct 2015 | Annual Celebration events | Publicity and Showcasing | As one makes advances towards their goal, aspirations need to be redefined to reenergise the frontline staff and avoid complacency. | Refresh the plan annually |
