# Supplementary material for: PROGRESS: the PROMISE governance framework to decrease coercion in mental healthcare
Source: BMJ Open Qual. 2018 Jul 16;7(3):e000332. doi: 10.1136/bmjoq-2018-000332 (PMC6059331; doi:10.1136/bmjoq-2018-000332)
Supplement: Supplementary data [file bmjoq-2018-000332supp007.docx]

Supplementary Table 4: Number of restraints per person from April 2015 to March 2017

| Number of restraints per person | 1 | 2 | 3 | 4 | 5 | 6 | 7 | 8 | 10 | 12 | 41 | 87 | Total |
| --- | --- | --- | --- | --- | --- | --- | --- | --- | --- | --- | --- | --- | --- |
| Individuals restrained | 104 | 28 | 11 | 12 | 5 | 1 | 3 | 1 | 1 | 1 | 1 | 1 | 169 |
